# P-1089. Pharmacokinetics of Rifaquizinone in Rats and Dogs Following Intra-Articular Administration

**DOI:** 10.1093/ofid/ofae631.1277

**Published:** 2025-01-29

**Authors:** Huan Wang, Qin Cheng, Haiwei Xiong, Zhenkun Ma

**Affiliations:** TenNor Therapeutics (Suzhou) Ltd, Suzhou, Jiangsu, China(People's Republic); TenNor Therapeutics (Suzhou) Ltd, Suzhou, Jiangsu, China(People's Republic); WuXi AppTec (Shanghai) CO., Ltd., Shanghai, Shanghai, China; TenNor Therapeutics, Suzhou Industrial Park, Jiangsu, China (People's Republic)

## Abstract

**Background:**

Rifaquizinone (RFQ, TNP-2092) is a novel multitargeting drug conjugate in development for the treatment of prosthetic joint infections (PJIs). RFQ exerts its antibacterial activity by inhibiting RNA polymerase, DNA gyrase, and topoisomerase IV. The current studies investigate the pharmacokinetics (PK) of RFQ following intra-articular (IA) administration to rats and dogs.

**Figure:**
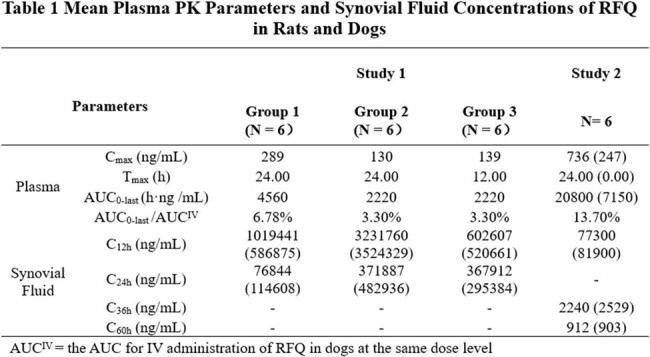

**Methods:**

In the first study, 18 male rats were assigned into Group 1-3 and received a single dose of 10 mg/kg RFQ formulated in 5% (w/v) glucose, saline, or 4% (w/v) Solutol HS15 in 5% (w/v) glucose, respectively. In the second study, six (3/sex) beagle dogs received a single dose of 3 mg/kg RFQ in 5% (w/v) glucose on the left leg and an equal volume of vehicle on the right leg. Blood and synovial fluid samples were collected and concentrations of RFQ were determined by liquid chromatography tandem mass spectrometry.

**Results:**

Following a single dose of IA administration of 10 mg/kg RFQ in rats, the maximum plasma concentrations (C_max_) were 289, 130 and 139 ng/mL in Group 1, Group 2 and Group 3, respectively. The mean synovial fluid concentrations of RFQ reached 76,844, 371,887 and 367,912 ng/mL at 24 hour after dosing for Group 1, Group 2, and Group 3, respectively. Following a single dose of IA administration of 3 mg/kg of RFQ in dogs, the C_max_ in plasma was 736 ng/mL, while the mean synovial fluids concentrations at 12 and 36 hours post dosing were 77,300 and 2,240 ng/mL, respectively. The synovial fluid concentration were 38.7 and 1.12 fold higher than the minimum biofilm bactericidal concentration inhibit 90% bacterial growth (MBBC_90_) for S. aureus (MBBC_90_ = 2,000 ng/mL) and 309 and 8.96 fold higher than the MBBC_90_ for S. epidermidis (MBBC_90_ = 250 ng/mL).

**Conclusion:**

The 5% (w/v) glucose appeared to be the best vehicle for IA administration of RFQ based on the tolerability and elimination profile. RFQ reached a high local synovial fluid concentration significantly higher than the MBBC_90_ for S. aureus and S. epidermidis for a prolonged period of time, while maintaining a low systemic exposure. The current studies supporting further evaluation of RFQ for the treatment of PJI via IA administration.

**Disclosures:**

**Huan Wang, PhD**, TenNor Therapeutics (Suzhou) Ltd: Employee **Qin Cheng, Master**, TenNor Therapeutics (Suzhou) Ltd: Employee **Haiwei Xiong, Master**, TenNor Therapeutics (Suzhou) Ltd: Investigator **Zhenkun Ma, PhD**, TenNor Therapeutics (Suzhou) Ltd: Employee

